# BMI and pelvimetry help to predict the duration of laparoscopic resection for low and middle rectal cancer

**DOI:** 10.1186/s12893-022-01840-4

**Published:** 2022-11-21

**Authors:** Wenhao Teng, Jingfu Liu, Meimei Chen, Weidong Zang, Aiwen Wu

**Affiliations:** 1 Department of Gastrointestinal Surgery, Clinical Oncology School of Fujian Medical University, Fujian Cancer Hospital, Fuzhou, 350014 China; 2 Department of Blood Transfusion, Clinical Oncology School of Fujian Medical University, Fujian Cancer Hospital, Fuzhou, China; 3 Unit III, Gastrointestinal Cancer Center, Key Laboratory of Carcinogenesis and Translational Research (Ministry of Education/Beijing), Peking University Cancer Hospital & Institute, Beijing, 100142 China

**Keywords:** Rectal cancer, Pelvimetry, Laparoscopic resection, Operative time, Total mesorectal excision

## Abstract

**Background:**

In rectal cancer surgery, recent studies have found associations between clinical factors, especially pelvic parameters, and surgical difficulty; however, their findings are inconsistent because the studies use different criteria. This study aimed to evaluate common clinical factors that influence the operative time for the laparoscopic anterior resection of low and middle rectal cancer.

**Methods:**

Patients who underwent laparoscopic radical resection of low and middle rectal cancer from January 2018 to December 2020 were retrospectively analyzed and classified according to the operative time. Preoperative clinical and magnetic resonance imaging (MRI)-related parameters were collected. Logistic regression analysis was used to identify factors for predicting the operative time.

**Results:**

In total, 214 patients with a mean age of 60.3 ± 8.9 years were divided into two groups: the long operative time group (n = 105) and the short operative time group (n = 109). Univariate analysis revealed that the male sex, a higher body mass index (BMI, ≥ 24.0 kg/m^2^), preoperative treatment, a smaller pelvic inlet (< 11.0 cm), a deeper pelvic depth (≥ 10.7 cm) and a shorter intertuberous distance (< 10.1 cm) were significantly correlated with a longer operative time (*P* < 0.05). However, only BMI (OR 1.893, 95% CI 1.064–3.367, *P* = 0.030) and pelvic inlet (OR 0.439, 95% CI 0.240–0.804, P = 0.008) were independent predictors of operative time. Moreover, the rate of anastomotic leakage was higher in the long operative time group (*P* < 0.05).

**Conclusion:**

Laparoscopic rectal resection is expected to take longer to perform in patients with a higher BMI or smaller pelvic inlet.

## Introduction

Colorectal cancer is the third most common cancer and the second leading cause of cancer-related death among all malignant tumors in the world [1]. Total mesorectal excision (TME) is the standard of radical surgery for rectal cancer and directly affects the local recurrence and overall survival [2, 3]. Laparoscopic rectal resection has been shown to be comparable to open surgery in terms of safety and long-term prognosis but is less invasive and allows faster recovery [4–7]. Especially in patients with low and middle rectal cancer, laparoscopic surgery provides a better view of the surgical field and finer anatomy [8], which is conducive to improving the quality of TME. Nevertheless, laparoscopic TME can be challenging in some patients with a narrow pelvis [9].

In addition to the surgical technique, surgical difficulty may be affected by body mass index (BMI), sex, tumor location, tumor size, and pelvic size [10]. Preoperative magnetic resonance imaging (MRI) can be used to determine the depth of tumor invasion, circumferential resection margin (CRM) involvement, vascular invasion, and lymph node enlargement; as such, it is an essential tool for determining the optimal treatment strategy for rectal cancer. Moreover, an MRI evaluation of pelvic shape and tumor position is also helpful for surgical resection planning. A recent meta-analysis showed that MRI-based pelvic measurements could effectively predict the surgical difficulty of TME [11]. However, the pelvic measurement parameters reported in the relevant literature are inconsistent. Previous studies mostly used the criteria proposed by Escal [12] for evaluation, but we found they had some limitations rendering them unsuitable for all centers.

The operative time was utilized as the primary indicator of intraoperative difficulty, as in previous studies [13, 14]. In this study, a group analysis was conducted based on the median operative time of different surgeons. This approach could reflect the complexity of surgery more objectively. We focused on evaluating common clinical factors, including pelvic parameters measured by MRI, influencing the operative time for the laparoscopic anterior resection of low and middle rectal cancer.

## Patients and methods

### Patients

Between January 2018 and December 2020, consecutive patients with rectal cancer who underwent laparoscopic radical resection at Peking University Cancer Hospital were identified. Patients with mid/low rectal adenocarcinoma (within 10 cm of the anal verge) were included. Patients who had undergone a more extensive surgery, including pelvic exenteration, combined evisceration or lateral pelvic lymph node dissection, abdominal perineal resection (APR), or transanal TME (TaTME), and those with insufficient preoperative MRI data were excluded. This study was approved by the Medical Ethics Committee of Peking University Cancer Hospital.

### Study design

All surgeons belonged to the same department and received structured training on laparoscopic surgery. Each patient was placed in the lithotomy position under general anesthesia. Five ports were used to conduct laparoscopic tumor-specific TME. The rectum was transected at least 2 cm distal to the tumor, while mesorectal tissue was resected at a position at least 5 cm distal from the tumor or to the levator ani. Then, end-to-end anastomosis was completed with the double-stapling technique. All operations were performed by experienced surgeons who had completed over 100 laparoscopic rectal cancer surgeries. Moreover, the surgical teams were relatively stable.

In this study, some patients underwent ileostomy simultaneously with resection. To reduce the impact of this extra procedure, the operative time for these patients was defined as the originally recorded time minus 15 min. Then, the median operative time of each surgeon was set as the cutoff value. Patients whose operative time was longer than the cutoff value of the corresponding surgeon were assigned to the long operative time (LOT) group, and the others to the short operative time (SOT) group. Then the clinical data were compared.

### Pelvic MRI measurements

**Fig. 1:**
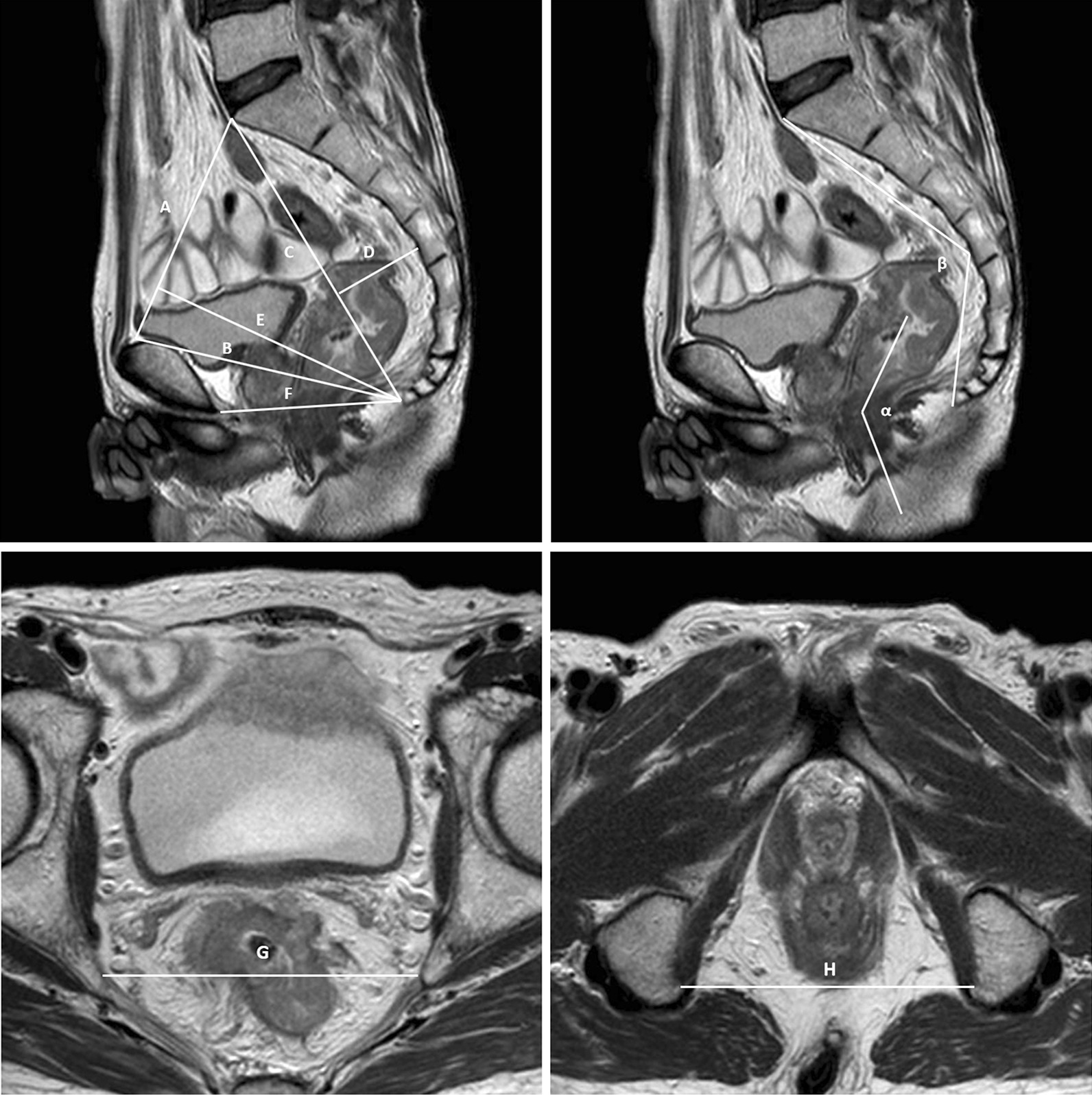
MRI-based pelvimetry. A: pelvic inlet; B: pubococcygeal distance; C: pelvic length; D: sacral depth; E: pelvic depth; F: pelvic outlet; G: interspinous distance; H: intertuberous distance; α: anorectal angle; β: sacrococcyx angle

All patients analyzed underwent an abdominopelvic MRI examination before the operation. T2-weighted imaging (T2WI) was used to measure the pelvimetric parameters, including eight on the sagittal view and two on the transverse view (Fig. 1). The following measurements were obtained: (1) pelvic inlet: distance from the sacral promontory to the superior aspect of pubic symphysis; (2) pubococcygeal distance: distance between the tip of the coccyx and superior aspect of pubic symphysis; (3) pelvic length: distance from the tip of the coccyx to the sacral promontory; (4) sacral depth: perpendicular distance from the sacrococcygeal line to the deepest point of the sacrococcygeal hollow; (5) pelvic depth: perpendicular distance from the tip of the coccyx to the pelvic inlet line; (6) pelvic outlet: distance from the tip of the coccyx to the inferior aspect of the pubic symphysis; (7) interspinous distance: distance between the tips of the ischial spines; (8) intertuberous distance: distance between the lowest points of the ischial tuberosities; (9) anorectal angle: angle between the rectum and anal canal; and (10) sacrococcygeal angle: angle between the deepest point of the sacrococcygeal hollow to the sacral promontory and the tip of the coccyx.

### Outcome measures

The primary outcome was predictors of the operative time. The secondary outcome was the early morbidity rate within 30 days after surgery.

### Statistical analysis

The statistical analysis was performed using SPSS version 22.0. Categorical variables are expressed as the mean ± standard deviation and were evaluated using a χ^2^ test or Fisher’s exact test. Continuous variables are presented as numbers and percentages and were evaluated using an independent t test or the Mann–Whitney U test. Logistic regression was performed to determine predictors of the operative time. The cutoff points for MRI pelvimetric parameters were defined as their median values. A two-sided P < 0.05 was considered statistically significant.

## Results

### Patient characteristics

**Fig. 2:**
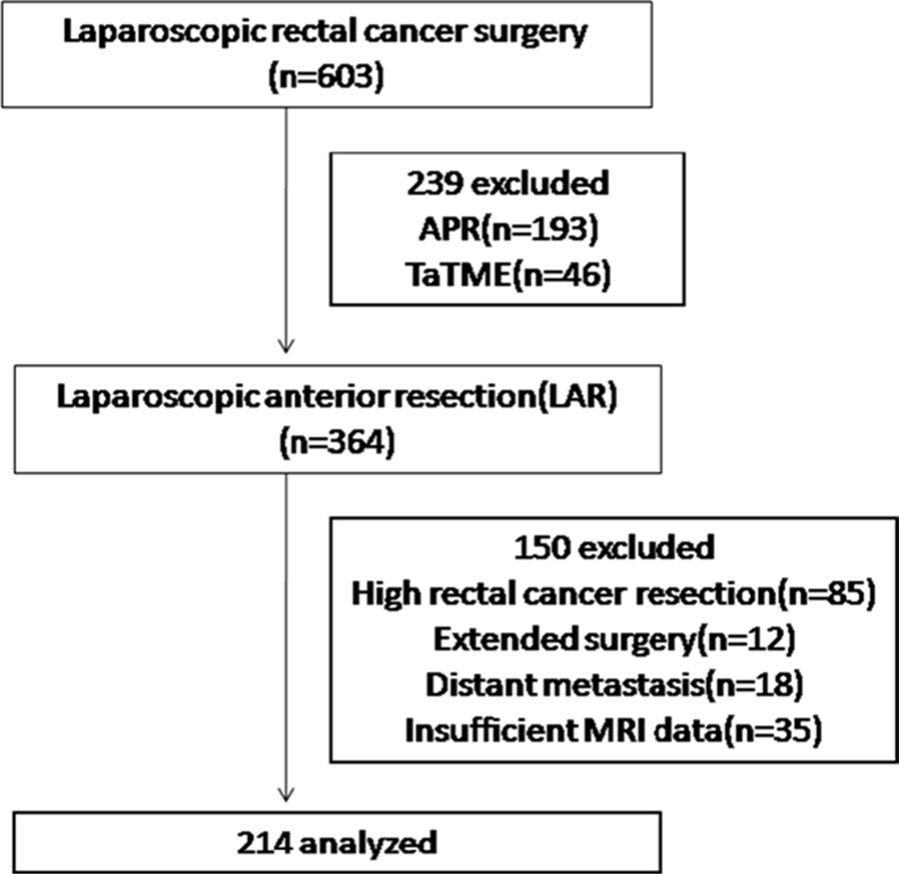
Flow chart of patient selection

Six hundred and three patients with rectal cancer who underwent laparoscopic radical resection were identified. And 214 patients with a mean age of 60 years were enrolled in the final analysis (Fig. 2). Most patients were male (63.6%). The median BMI, distance from the anal verge, and tumor size were 24.1 kg/m^2^, 7.0 cm, and 3.0 cm, respectively. A total of 53.3% of patients received neoadjuvant treatment, and 59.8% underwent ileostomy. Among all patients, 28 patients (13.1%) had a prior history of abdominal surgery.

### Comparison of parameters between the SOT and LOT groups

Based on the above definitions, 109 and 105 cases were included in the SOT and LOT groups, respectively, with a median operative time of 119.3 ± 25.0 min and 170.3 ± 30.8 min, respectively (P < 0.05). As illustrated in Table 1, the patients in the LOT group were mostly male, had a higher BMI, more preoperative treatment, a smaller pelvic inlet, a shorter intertuberous distance, and a deeper pelvis (P < 0.05) (Table 1).

**Table 1:** Comparison of parameters between the SOT and LOT groups

Factors	Surgery type		P value
	SOT (n = 109)	LOT (n = 105)	
Age (years)	59.9 ± 9.4	60.8 ± 8.4	0.483
Sex (male: female)	62: 47	74: 31	0.039^a^
BMI (kg/m^2^)	23.6 ± 2.8	24.5 ± 2.8	0.024
Comorbidity	29(26.6)	32(30.5)	0.531^a^
History of abdominal surgery	17(15.6)	11(10.5)	0.267^a^
Preoperative treatment	50(45.9)	64(61.0)	0.027^a^
Distance from the anal verge (cm)	7.2 ± 2.1	6.7 ± 2.4	0.127
Tumor size (cm)	2.9 ± 1.7	3.1 ± 1.8	0.563
Pathological stage (0 + I: II + III)	51:58	36:69	0.063^a^
Pelvic inlet (cm)	11.3 ± 1.1	10.8 ± 1.1	0.001
Pubococcygeal distance (cm)	11.8 ± 1.0	12.0 ± 0.9	0.382
Pelvic length (cm)	12.5 ± 1.3	12.6 ± 1.1	0.785
Sacral depth (cm)	3.8 ± 0.6	3.8 ± 0.5	0.673
Pelvic depth (cm)	10.6 ± 1.0	10.9 ± 0.9	0.025
Pelvic outlet (cm)	8.0 ± 0.9	7.9 ± 0.8	0.568
Interspinous distance (cm)	9.7 ± 1.3	9.5 ± 1.1	0.413^b^
Intertuberous distance (cm)	10.3 ± 1.5	9.9 ± 1.4	0.048
Anorectal angle (°)	119.6 ± 11.7	119.4 ± 15.5	0.895^b^
Sacrococcygeal angle (°)	114.2 ± 10.3	115.1 ± 8.3	0.460^b^

*LOT* long operative time; *SOT* short operative time; *BMI* body mass index

^a^χ^2^ test

^b^Mann–Whitney U test

### Predictors of operative time

Univariate analysis revealed that the male sex, a higher BMI (≥ 24.0 kg/m^2^), preoperative treatment, a smaller pelvic inlet (< 11.0 cm), a deeper pelvic depth (≥ 10.7 cm) and a shorter intertuberous distance (< 10.1 cm) were significantly correlated with a longer surgical duration (P < 0.05). However, only BMI (OR 1.893, 95% CI 1.064–3.367, P = 0.030) and pelvic inlet (OR 0.439, 95% CI 0.240–0.804, P = 0.008) were independent predictors of operative time (Table 2).

**Table 2:** Logistic regression analysis of predictors related to operative time

Factors	Univariate analysis		Multivariate analysis	
	OR (95% CI)	P value	OR (95% CI)	P value
Sex (male vs. female)	0.553 (0.314–0.973)	0.040	1.074 (0.449–2.569)	0.873
BMI (< 24.0 vs. ≥ 24.0 kg/m^2^)	1.979 (1.148–3.410)	0.014	1.893 (1.064–3.367)	0.030
Preoperative treatment (yes vs. no)	0.543 (0.315–0.935)	0.028	0.564 (0.317–1.003)	0.051
Pelvic inlet (< 11.0 vs. ≥ 11.0 cm)	0.371 (0.213–0.645)	0.000	0.439 (0.240–0.804)	0.008
Pelvic depth (< 10.7 vs. ≥ 10.7 cm)	1.837 (1.067–3.161)	0.028	1.450 (0.787–2.670)	0.233
Intertuberous distance (< 10.1 vs. ≥ 10.1 cm)	0.543 (0.315–0.935)	0.028	0.667 (0.303–1.469)	0.315

### Complications in different groups

Complications in the different groups were analyzed. There were no differences in pulmonary infection, pelvic infection, postoperative ileus, or anastomotic bleeding. However, the anastomotic leakage rate was higher in the LOT group (P < 0.05) (Table 3).

**Table 3:** Complications in different groups

Complications	Surgery type		P value
	SOT (n = 109)	LOT (n = 105)	
Pulmonary infection	1 (0.9)	0 (0)	1.000^a^
Pelvic infection	2 (1.8)	0 (0)	0.498^a^
Postoperative ileus	3 (2.8)	2 (1.9)	1.000^a^
Anastomotic bleeding	1 (0.9)	0 (0)	1.000^a^
Anastomotic leakage	3 (2.8)	10 (9.5)	0.047^a^
Total	10 (9.2)	12 (11.4)	0.587^b^

*LOT* long operative time; *SOT* short operative time

^a^Fisher’s exact test

^b^χ^2^ test

## Discussion

Recent studies have shown that several parameters are associated with the surgical difficulty of laparoscopic anterior resection for low and middle rectal cancer, but the results are inconsistent [15–18]. The present study demonstrated that a higher BMI and smaller pelvic inlet could help predict the duration of surgery, which might be helpful for preoperative assessment.

Operating rooms currently account for 35–40% of hospital costs and 60–70% of hospital revenues [19]. The improvement of operating room productivity has an important impact on the financial performance and ultimately the ethical performance of the hospital [20]. Therefore, hospital management focuses on the effectiveness of schedules and plans. In our study, the results showed that we could use BMI and pelvic inlet to predict the operative time. These are two common parameters that can be easily obtained. We believe that a simple preoperative evaluation to measure BMI and pelvic inlet can help to determine more effective operating room arrangements, especially in centers that are short on surgical resources [21].

The criteria for grading surgical difficulty proposed by Escal [12] include duration of surgery > 300 min, conversion to open procedure, use of transanal dissection, postoperative hospital stay > 15 days, blood loss > 200 ml, and morbidity (grades II and III). The surgical difficulty grade ranged from 0 to 12, and patients scoring six or higher were considered to have high surgical difficulty. However, we found that the critical value of the criteria varies significantly from center to center. Yamamoto [15] analyzed the data of 121 patients undergoing minimally invasive rectal surgery and found that the median blood loss was only 30 ml, which is lower than the 200 ml proposed by Escal [12]. Therefore, Yamamoto [15] changed the threshold for blood loss to 100 ml. Moreover, the median operative time and postoperative hospital stay were 310 min and 18 days, respectively, so those criteria were also adjusted accordingly. However, in the studies by Sun [18] and Chen [16], the average postoperative hospital stay was 8.0 days and 7.7 days, respectively. As a result, these authors adjusted the standard critical value of postoperative hospital stay to 7 days for analysis. In addition, they defined difficult operations with an overall score greater than 3 points, rather than the six used in the previous study. Although different centers reported adopting Escal’s grading standards, most of them adopted adjusted standards, indicating that there are still limitations inherent to these grading criteria. In our opinion, the criteria can be influenced by many factors, such as the surgeon’s style and behavior, the availability of rapid rehabilitation, and medical resources. Therefore, we thought none of the criteria could exactly reflect the surgical difficulty until now. In this study, we compared differences in the operative time, which could be accurately obtained, between different patients. Moreover, we proposed that individual grouping based on the operative times of different surgeons would be more beneficial to make the results more objective and repeatable.

Anastomotic leakage is one of the most common postoperative complications of surgery for rectal cancer and can prolong the hospital stay, delay the adjuvant treatment, increase the financial burden, and even lead to death in serious cases [22]. In addition, anastomotic leakage has been found to be related to increased local recurrence and decreased overall survival, cancer-specific survival, and disease-free survival [23]. This study showed that the rate of anastomotic leakage after rectal cancer surgery was higher in the LOT group, which is consistent with results reported by Qu [24]. Therefore, these patients merit more attention in clinical work and timely monitoring when necessary. Moreover, the operative time was found to be related to obesity and to a small pelvic inlet. Obese patients have a thick mesorectum, which leads to a relatively narrow pelvic cavity that makes the surgery difficult. During the procedure, to fully expose the surgical field, repeated strong pulling of the proximal intestinal tube and tissues surrounding the site of anastomosis would increase additional damage, resulting in poor postoperative anastomotic healing. In addition, the small pelvic inlet makes it challenging to insert the stapler into the deep pelvis or necessitates more stapler firings for rectal transection, both of which are risk factors for anastomotic leakage [25].

Obesity has been found to be associated not only with increases in operative time and blood loss [14, 26, 27] but also with higher rates of anastomotic leakage, surgical-site infection (SSI), urinary tract infection (UTI), sepsis, and venous thromboembolism (VTE) [28–30]. The increased visceral obesity volume and mesenteric fat area (MFA) in obese patients makes performing laparoscopic surgery for rectal cancer a unique challenge [31]. In this study, the patient BMI ranged from 16.2 to 31.4 kg/m^2^, with a mean 24.1 kg/m^2^, which is lower than that in Western populations. Nevertheless, our results agreed with those of previous reports that found a positive association between BMI and operative time [13, 32].

On the other hand, studies have shown that the bony structure of the pelvis, such as the depth and length of the sacrum, the pelvic inlet and outlet, and the angle of the pelvis, are independent predictors of the duration of surgery and can be used as surrogate markers of TME difficulty [11, 15, 33]. In our study, the univariate analysis results showed differences in three bone indices, i.e., the pelvic inlet, ischial intertuberous diameter, and pelvic depth, suggesting that a deep and narrow pelvis did affect the duration of surgery. Restricted working space directly affects how difficult it is to perform surgery safely and quickly; in addition, visibility and coordination are required for surgery in these spaces to be optimized. Moreover, multivariate analysis showed that the pelvic inlet was an independent risk factor; thus, this metric merits special attention in rectal resection patients. In our retrospective review of surgical videos of some patients who underwent prolonged surgery, we found that several techniques might help to reduce the operative time, such as suspending the uterus or peritoneal reflection, lifting the upper rectum with a string, and wiping the lenses with iodophor to prevent fog. However, what mattered most was teamwork. We believe that these issues should not be a major problem for professional surgical teams because they have more experience in creating suitable surgical areas and are able to identify and anatomize structures even in a restricted pelvic working space.

Not surprisingly, there are some limitations to this study. This was a retrospective analysis, and the operative time was measured from the beginning of anesthesia to the end of surgery, rather than as the pelvic anatomy time. However, the inclusion criteria were strict, and cases of high rectal cancer, lateral lymph node dissection, multivisceral resection, and transanal dissection were excluded to minimize the influence of confounding factors. In addition, pelvic measurements in this study were made by a single observer; as such, quantification of interobserver variability could not be performed.

## Conclusions

In conclusion, our findings indicate that a higher BMI and smaller pelvic inlet are significantly associated with a longer operative time. These two parameters, BMI and pelvic inlet, are helpful for predicting the duration of TME for low and middle rectal cancer and should be evaluated preoperatively.

## Data Availability

The datasets used and/or analyzed during the current study are available from the corresponding author.

## Abbreviations

TME: Total mesorectal excision
CRM: Circumferential resection margin
APR: Abdominal perineal resection
TaTME: Transanal total mesorectal excision
LOT: Long operative time
SOT: Short operative time
BMI: Body mass index
SSI: Surgical-site infection
UTI: Urinary tract infection
VTE: Venous thromboembolism
MFA: Mesenteric fat area
